# Value of CCNA1 Promoter Methylation in Triaging ASC-US Cytology

**DOI:** 10.31557/APJCP.2020.21.2.473

**Published:** 2020

**Authors:** Shina Oranratanaphan, Kewalin Kobwitaya, Wichai Termrungruanglert, Surang Triratanachat, Nakarin Kitkumthorn, Apiwat Mutirangura

**Affiliations:** 1 *Department of Obstetrics and Gynecology, *; 3 *Center of Excellence in Molecular Genetics of Cancer and Human Diseases, Department of Anatomy, Faculty of Medicine, Chulalongkorn University,*; 2 * Department of Oral and Maxillofacial Pathology, Faculty of Dentistry, Mahidol University, Bangkok Thailand. *

**Keywords:** CCNA1, ASC-US, accuracy, diagnostic value, CIN2+

## Abstract

**Background:**

Using HPV testing to triage ASC-US still has some problems of unnecessary colposcopy in many cases. A previous study reported that methylation of CCNA1, a tumor suppressor gene, can differentiate between low and high grade lesions. This study was designed to evaluate the diagnostic values and application of CCNA1 methylation in the patients with ASC-US group.

**Materials and Methods:**

Cross sectional analytic study was conducted in the patients with ASC-US cytology. HPV DNA testing and CCNA1 promoter methylation testing were performed. The patients were sent for colposcopic examination and biopsy. Biopsy results were considered as gold standard. Diagnostic test of HPV test and CCNA1 methylation test were calculated for sensitivity, specificity, negative predictive value (NPV), positive predictive value (PPV), likelihood ratio for test positive and negative and 95% confidence interval.

**Results:**

One hundred and seventy patients were enrolled. Mean age was 39.7 years old. HR-HPV was positive in 70% of the patients. HPV type 16, type 18 and non-16,18 were 12.4%, 4.7% and 42.4%, respectively. CIN2+ were found in 12.4% (21 cases). CCNA1 promoter methylation was positive in 5 cases. CCNA1 had high specificity 99.3%, NPV 89.2% and PPV 80% in detection of CIN2+ but sensitivity was 19%. Likelihood ratio for positive test was 28.4 and likelihood ratio for negative test was 0.8. HPV test had sensitivity of 90.5% and NPV of 95.9% but low specificity and PPV as 31.5% and 15.7%, respectively.

**Conclusion:**

CCNA1 promoter methylation testing had very high specificity, likelihood ratio for the positive test and PPV (99.3%, 28.4 and 80.0, respectively). Therefore, CCNA1 promoter methylation test may be used in the HPV DNA positive cases to classify the urgency of colposcopy and the colposcopist should pay more attention to CCNA1 positive patients because of their higher chance to identify the significant lesions.

## Introduction

Cervical cancer is the third most common gynecological cancer worldwide (Ferlay et al., 2015). The incidence of cervical cancer is 14 per 100,000-person year (Pongnikorn et al., 2015). It is the second most common cancer in Thai women. Atypical squamous cell of undetermined significant (ASC-US) cytology is the minor cytologic abnormality. Incidence of invasive cancer in ASC-US is low and varies from 0.1 to 1% (Sundstrom et al., 2017; Tokmak et al., 2014). According to American Society for Colposcopy and Cervical Pathology (ASCCP) guideline 2012 (Saslow et al., 2012), there are two options to manage ASC-US including follow up and reflect HPV testing. HPV testing is used to triage because the risk of cervical cancer in HPV negative patients is extremely low (Kjaer et al., 2010). Therefore, HPV negative patients should not be sent for colposcopy (de Sanjose et al., 2010). However, HPV testing has low specificity and low positive predictive value (PPV) (25.1% and 17.5%, respectively) (Ko et al., 2006). Use of HPV testing still has some problems because positive rate of HPV testing in ASC-US patients are as high as 31.5% while the high grade lesion in ASC-US is only 5-10%. Therefore, HPV testing results in unnecessary colposcopy in many cases.

Some studies reported that CyclinA1gene which is a tumor suppressor gene that was activated after E6 and E7 protein of HPV embedded in the host cells leaded to progress into cervical cancer. Some studies reported that the CCNA1 methylation was found in high grade lesions and cervical cancer but no CCNA1 promoter methylation in normal cervixes (Chujan et al., 2014; Yang et al., 2010; Yanatatsaneejit et al., 2011). The previous study reported that CCNA1 methylation in normal cervixes, LSIL and HSIL are different (0% vs. 2.88% vs. 83.33%, respectively) (Khunamornpong et al., 2014). Therefore, CCNA1 methylation may be used to triage atypical squamous cell of undetermined significance (ASC-US) (Stoler et al., 2011). This study was designed to evaluate the diagnostic values of CCNA1 methylation in the patients with ASC-US group and compare to HPV DNA test.

## Materials and Methods

This cross sectional analytic study was conducted in the patients with ASC-US cytology. The ASC-US specimens were reviewed by cytopathologists at King Chulalongkorn Memorial Hospital (KCMH). This study protocol has been approved by the Institutional Review Board (IRB), Faculty of Medicine, Chulalongkorn University (IRB No.603/59). 

All female patients with ASC-US cytology who came to colposcopy clinic at KCMH between February 2017 and January 2018 were recruited in this study. The patients who were performed Pap test from other hospitals were excluded. The participants signed their inform consent forms voluntarily. The process of cytologic specimen collection was done in the routine cervical cancer screening program by the residents, fellows and staff at KCMH. The collected cytologic specimen from BD SurePath™ liquid-based Pap test was sent for HPV DNA and CCNA1 promoter methylation testing. The cases with ASC-US cytology were sent to the colposcopy clinic for colposcopic examination and biopsy was performed at the most severe lesions. In case that no lesion was detected, random biopsy was performed. 

*HPV DNA Test*

HPV DNA test was performed with Cobas® HPV testing, fully automated test based on extraction of the HPV and cellular DNA then followed by real time PCR to detect 14 high risk HPVs which consisted of 16, 18 and other high risk types (31, 33, 35, 39, 45, 51, 52, 56, 58, 59, 66, and 68). After that, the specimens were sent to CCNA1 promoter methylation testing at our lab. 

*DNA bisulfite modification*

DNA concentration was measured by nanodrop and subsequently adjusted to 750 ng/µl. Bisulfite treatment to 20 µl of each sample was performed by using the EZ DNA Methylation Kit (Zymo Research). The converted DNA solutions were eluted in 20 µl of M-Elution Buffer and stored below -20°C for subsequent use.

*CCNA1 real-time PCR*

To detect CCNA1 promoter methylation, we designed two TaqMan™. probe real-time PCRs. The CCNA1 methylation set was composed of the forward primer (5’ GGTAGGAAGAGTAGGTGTGTG 3’), reverse primer (5’ ACAACCCCTAACAACCCCCTCTAA 3’) and probe (FAM-5’GGGTTAGAGTGGGTAG 3’-BHQ). The Beta actin set primers were designed in the area of no CpG island to serve as internal control. The Beta actin set consisted of forward primer, reverse primer and probe. Both PCR reactions were prepared in a volume of 20 µL containing 10 µL of 2X TaqMan GTXpress real-time PCR master mix, 0.4 µL of 10 µmoles of each primer as well as probes and 2 µL of bisulfite-treated DNA template; the remaining volume was adjusted by adding milliQ DNase-free sterile water. Real-time PCRs were performed in duplicates using Applied Biosystem^®^ 7500 Real-Time PCR System (Thermo Scientific™, Waltham, MA, USA). The PCR conditions were first denaturation at 95°C for 2 minutes then go after with 40 cycles as follows: denaturation at 95°C 15 seconds, followed by annealing at 60°C for 30 seconds. Negative control (dH20) and positive control (universal human methylated DNA (EpiTect^®^PCR control kit, Qaigen, USA)) were included in each PCR. A melting curve was generated to determine the specificity of the primers. Later, the threshold cycle (Ct) of the amplified methylation products was detected. The results of all samples must have Beta actin products as internal control. Data analysis reported positive if present of CCNA1 methylation product and reported negative if no CCNA1 methylation.

*Gold standard*

The gold standard for diagnosis was the most severe pathologic results from colposcopic directed biopsy. Colposcopists in this study had to pass the colposcopic training course and had to pass the 50 validated cases before performed colposcopic examination in this study. Colposcopic examination reports were recorded. Colposcopic impression which meant the opinion of the colposcopist whether the lesion was high grade or low grade before biopsy. The biopsy results were reviewed by pathologists and recorded.

*Statistical analysis*

Statistical analysis was performed using SPSS software version 22.0 (IBM Corp, Armonk, N.Y., USA). Demographic data were analyzed by descriptive method. Diagnostic test was calculated both HPV test and CCNA1 methylation test for sensitivity, specificity, negative predictive value (NPV), positive predictive value (PPV) and 95% confidence interval. Sample size was calculated by infinite population proportion formula based on sensitivity of CCNA1 from the previous study which reported 83% (Chujan et al., 2014). Therefore, 170 participants were required in this study. 

## Results

All of the 211 patients of ASC-US cytology who underwent colposcopic directed biopsy at colposcopy clinic at King Chulalongkorn Memorial Hospital (KCMH), Bangkok, Thailand from February 2017 to January 2018. Forty-one cases were excluded because the cases had undergone Pap smear test from other hospitals. Accordingly, the remaining 170 cases were included to performed HPV DNA and CCNA1 methylation testing.

Demographic data are shown in Table1. Mean age of the subjects was 39.7 years old, standard deviation (SD) was 11.1. Most subjects were premenopausal status 80% (136 cases) and had more than 1 partner 52.9% (90 cases). Majority of the colposcopic impressions which meant the opinion of colposcopist before the final pathologic results report were low-grade lesion 70.6% (120 cases) and no lesion was found 10.6% (18 cases). High-grade lesion by colposcopic impression was only 9.4% (16 cases). Seventy percent of the cases had high risk HPV infection consisted of HPV type 16, type 18 and other high risk types were 12.4%, 4.7% and 42.4%, respectively. The final pathology after colposcopic biopsy was found benign lesion (HPV infection or chronic cervicitis) 67.1% (114 cases), CIN1 20.6% (35 cases) and CIN2+ 12.4% (21 cases). CIN 2+ cases consisted of 7cases of CIN2, 13 cases of CIN 3 and 1 case of AIS. There was no invasive lesion found in ASC-US cytology in this study.

CCNA1 promoter methylation was test in 170 specimens and positive test was found in 5 cases. The positive cases consisted of CIN2 (2 cases) CIN3 (2 cases) and condyloma (1 case); details are shown in Table 2. Diagnostic value of the CCNA1 promoter methylation test in ASC-US cytology is shown in Table 2, 3. CCNA1 had high specificity 99.3%, negative predictive value (NPV) 89.2% and positive predictive value (PPV) 80% in detection of CIN2+. However, sensitivity of the test was only 19%. Likelihood ratio for positive test was 28.4 which was very high and likelihood ratio for negative test was 0.8.

Results of HPV testing are shown in Table 2 and the diagnostic value of HPV test is shown in Table 3. We found HPV test had high sensitivity of 90.5% and NPV of 95.9% but low specificity and PPV as 31.5% and 15.7%, respectively (Table 3).

**Table 1:** Demographic Data of the ASC-US Population (N=170)

Character	N (%)
Age (years) (Mean + SD)	39.7 + 11.1
Menopause: N (%)	
Premenopause	136 (80%)
Postmenopause	34 (20%)
Partner (N (%))	
Single	80 (47.1%)
Multiple	90 (52.9%)
Colposcopic findings (N (%))	
No lesion	18 (10.6%)
HPV or condyloma	16 (9.4%)
Low grade lesion	120 (70.6%)
High grade lesion	16 (9.4%)
HPV DNA (N (%))	
Negative	51 (30%)
Type 16	21 (12.4%)
Type 18	9 (4.7%)
Non 16, 18, other high risk	72 (42.4%)
More than 1 type	18 (10.5%)
Histopathology (N (%))	
Benign (HPV, chronic cervicitis)	114 (67.1%)
CIN1	35 (20.6%)
CIN2+	21 (12.4%)

**Table 2:** CCNA1 Promoter Methylation and HPV Testing Results

Test	CIN2+	CIN1-	Total
CCNA1			
Positive	4	1	5
Negative	17	148	165
HPV			
Positive	19	102	121
Negative	2	47	49
Total	21	149	170

**Table 3:** Diagnostic Value for CCNA1 and HPV Testing

Diagnostic value	CCNA1	HPV
Sensitivity % (95%CI)	19.0 (7.0-40.0)	90.5 (71.1-97.4)
Specificity % (95%CI)	99.3 (96.3-99.8)	31.5 (24.6-39.4)
Accuracy % (95%CI)	89.4 (83.9-93.2)	38.8 (31.8-46.3)
PPV% (95%CI)	80.0 (37.5-96.4)	15.7 (10.3-23.2)
NPV % (95%CI)	89.2 (83.5-93.0)	95.9 (86.2-98.9)
LR for test positive	28.4	1.3
LR for test negative	0.8	0.3

## Discussion

The final outcomes of ASC-US in this study had high grade histology (CIN2+) 12.4%. This prevalence of high grade lesion in ASC-US is higher than the previous study in our institution in 2008-2012 which was 6.71% (Tantitamit et al., 2015). The different of prevalence may result from the widely use of reflect HPV based on ASCCP recommendation. Therefore, ASC-US with negative HPV was not sent to perform colposcopy. However, some of the patients insisted to perform colposcopy instead of HPV triage were also sent to colposcopic clinic. For those reason, the proportion of high grade histology in ASC-US in this study was higher than previous study. However, this prevalence concordance with the study in Northern part of Thailand that found high grade histology 10-20% in ASC-Us cytology (Khunamornpong et al., 2014). The colposcopic impression or the opinion of colposcopist in the ASC-US cytology was reported low-grade lesion 70.6% which concordance with the previous study which found low grade lesion in colposcopy in ASC-US cytology as 68.1% (Wentzensen et al., 2018). 

The diagnostic value of HPV testing in this study had high sensitivity 90.5% and high NPV 95.9% which was the high performance of screening test. Nevertheless, the specificity was low as 31.5%, which was concordance with the previous studies (Ko et al., 2006; Castle et al., 2015; Levi et al., 2003). Previous studies showed HPV testing had high sensitivity, low specificity and PPV in ASC-US cytology range 89.4-93.4%, 25.1- 59.3% and 17.5-20.3%, respectively (Ko et al., 2006; Castle et al., 2015). 

CCNA1 promoter methylation testing in this study had high specificity 99.3% which was the characteristic of the good diagnostic test. However, this test shown low sensitivity 19% which was too low to be a screening test. The data from the previous studies Chujan et al., (2014) and Kitkumthorn et al., (2006) showed that CCNA1 promotor methylation had high discrimination power to differential high grade lesion and invasive cervical cancer from low grade lesion (Chujan et al., 2014; Yang et al., 2009). The Likelihood ratio for the positive test of CCNA1 in our study was very high (28.4) which was represented a good diagnostic test. Moreover, positive predictive value of CCNA1 was also as high as 80.0 (37.5-96.4). High likelihood for the positive test result and PPV told us that if CCNA1 positive there was high chance to identify the high grade lesion in the patient. These results may be used in clinical application. For example, CCNA1 promoter methylation test may be use in the HPV DNA positive cases to classify the urgent of colposcopy in the long waiting time situation. In CCNA1 positive patients, urgent colposcopy may be required. In case that HPV and CCNA1 results were negative, the patients may be omitting colposcopy because of high NPV 94.7% in this test. From our study, colposcopic results showed that 5 cases of high grade lesions were underestimated from colposcopic impression. CCNA1 may help in this situation. In the patients who had CCNA1 positive, the colposcopist should pay more attention to identify the lesions because of the high likelihood ratio for positive test. 

Our strength of this study is the final pathological confirmation was performed in all cases which was the gold standard to evaluate the diagnostic values. There are some limitations in our study, however. First, this study had small amount of CIN2+ histopathology. Second, there was positive test of CCNA1 methylation only 3% which less than the other studies therefore the result still inconclusive to be used as triage model. For further study, we may increase the number of the patients with CIN2+ for evaluation. Hence, the result of diagnostic values and role of triage may be clearer.

In conclusion, CCNA1 promoter methylation test may not be a good screening test but CCNA1 may be useful when combined with HPV DNA test to allocate the urgency of colposcopy in the test positive patients and used as alert sign for carefully find the lesion when the test is positive. 

## Funding and support

This research was supported by a 2019 Research Chair Grant from the National Science and Technology Development Agency (NSTDA), Thailand, and the Anantara Siam Bangkok Hotel, Four Seasons Hotel Care for Cancer Fun Run in coordination with the Thai Red Cross Society.

## References

[B1] Castle PE, Eaton B, Reid J, Getman D, Dockter J (2015). Comparison of human papillomavirus detection by Aptima HPV and cobas HPV tests in a population of women referred for colposcopy following detection of atypical squamous cells of undetermined significance by Pap cytology. J Clin Microbiol.

[B2] Chujan S, Kitkumthorn N, Siriangkul S, Mutirangura A (2014). CCNA1 promoter methylation: a potential marker for grading Papanicolaou smear cervical squamous intraepithelial lesions. Asian Pac J Cancer Prev.

[B3] de Sanjose S, Quint WG, Alemany L (2010). Human papillomavirus genotype attribution in invasive cervical cancer: a retrospective cross-sectional worldwide study. Lancet Oncol.

[B4] Ferlay J, Soerjomataram I, Dikshit R (2015). Cancer incidence and mortality worldwide: sources, methods and major patterns in GLOBOCAN 2012. Int J Cancer.

[B5] Khunamornpong S, Settakorn J, Sukpan K (2014). Performance of HPV DNA testing with hybrid capture 2 in triaging women with minor cervical cytologic abnormalities (ASC-US/LSIL) in Northern Thailand. Asian Pac J Cancer Prev.

[B6] Kitkumthorn N, Yanatatsanajit P, Kiatpongsan S (2006). Cyclin A1 promoter hypermethylation in human papillomavirus-associated cervical cancer. BMC Cancer.

[B7] Kjaer SK, Frederiksen K, Munk C, Iftner T (2010). Long-term absolute risk of cervical intraepithelial neoplasia grade 3 or worse following human papillomavirus infection: role of persistence. J Nat Cancer Institut.

[B8] Ko V, Tambouret RH, Kuebler DL, Black-Schaffer WS, Wilbur DC (2006). Human papillomavirus testing using hybrid capture II with SurePath collection: initial evaluation and longitudinal data provide clinical validation for this method. Cancer.

[B9] Levi AW, Kelly DP, Rosenthal DL, Ronnett BM (2003). Atypical squamous cells of undetermined significance in liquid-based cytologic specimens: results of reflex human papillomavirus testing and histologic follow-up in routine practice with comparison of interpretive and probabilistic reporting methods. Cancer.

[B11] Saslow D, Solomon D, Lawson HW (2012). American society for colposcopy and cervical pathology, and American Society for Clinical Pathology screening guidelines for the prevention and early detection of cervical cancer. J Low Genit Tract Dis.

[B12] Stoler MH, Wright TC Jr, Sharma A (2011). High-risk human papillomavirus testing in women with ASC-US cytology: results from the ATHENA HPV study. Am J Clin Pathol.

[B13] Sundström K, Lu D, Elfström KM (2017). Follow-up of women with cervical cytological abnormalities showing atypical squamous cells of undetermined significance or low-grade squamous intraepithelial lesion: a nationwide cohort study. Am J Obstet Gynecol.

[B14] Tantitamit T, Termrungruanglert W, Oranratanaphan S (2015). Cost-effectiveness analysis of different management strategies for detection CIN2+ of women with Atypical squamous cells of undetermined significance (ASC-US) Pap smear in Thailand. Asian Pac J Cancer Prev.

[B15] Tokmak A, Guzel Al, Ozgu E (2014). Clinical significance of atypical squamous cells of undetermined significance in detecting preinvasive cervical lesions in post- menopausal Turkish women. Asian Pac J Cancer Prev.

[B16] Wentzensen N, Walker J, Smith K (2018). A prospective study of risk-based colposcopy demonstrates improved detection of cervical precancers. Am J Obstet Gynecol.

[B17] Yanatatsaneejit P, Mutirangura A, Kitkumthorn N (2011). Human papillomavirus’s physical state and cyclin A1 promoter methylation in cervical cancer. Int J Gynecol Cancer.

[B18] Yang N, Eijsink JJ, Lendvai A (2009). Methylation markers for CCNA1 and C13ORF18 are strongly associated with high-grade cervical intraepithelial neoplasia and cervical cancer in cervical scrapings. Cancer Epidemiol Biomarkers Prev.

[B19] Yang N, Nijhuis ER, Volders HH (2010). Gene promoter methylation patterns throughout the process of cervical carcinogenesis. Cell Oncol.

